# Assessing interactions between the associations of common genetic susceptibility variants, reproductive history and body mass index with breast cancer risk in the breast cancer association consortium: a combined case-control study

**DOI:** 10.1186/bcr2797

**Published:** 2010-12-31

**Authors:** Roger L Milne, Mia M Gaudet, Amanda B Spurdle, Peter A Fasching, Fergus J Couch, Javier Benítez, José Ignacio Arias Pérez, M Pilar Zamora, Núria Malats, Isabel dos Santos Silva, Lorna J Gibson, Olivia Fletcher, Nichola Johnson, Hoda Anton-Culver, Argyrios Ziogas, Jonine Figueroa, Louise Brinton, Mark E Sherman, Jolanta Lissowska, John L Hopper, Gillian S Dite, Carmel Apicella, Melissa C Southey, Alice J Sigurdson, Martha S Linet, Sara J Schonfeld, D Michal Freedman, Arto Mannermaa, Veli-Matti Kosma, Vesa Kataja, Päivi Auvinen, Irene L Andrulis, Gord Glendon, Julia A Knight, Nayana Weerasooriya, Angela Cox, Malcolm WR Reed, Simon S Cross, Alison M Dunning, Shahana Ahmed, Mitul Shah, Hiltrud Brauch, Yon-Dschun Ko, Thomas Brüning, GENICA Network, Diether Lambrechts, Joke Reumers, Ann Smeets, Shan Wang-Gohrke, Per Hall, Kamila Czene, Jianjun Liu, Astrid K Irwanto, Georgia Chenevix-Trench, Helene Holland, kConFab, AOCS, Graham G Giles, Laura Baglietto, Gianluca Severi, Stig E Bojensen, Børge G Nordestgaard, Henrik Flyger, Esther M John, Dee W West, Alice S Whittemore, Celine Vachon, Janet E Olson, Zachary Fredericksen, Matthew Kosel, Rebecca Hein, Alina Vrieling, Dieter Flesch-Janys, Judith Heinz, Matthias W Beckmann, Katharina Heusinger, Arif B Ekici, Lothar Haeberle, Manjeet K Humphreys, Jonathan Morrison, Doug F Easton, Paul D Pharoah, Montserrat García-Closas, Ellen L Goode, Jenny Chang-Claude

**Affiliations:** 1 Genetic and Molecular Epidemiology Group, Human Cancer Genetics Programme, Spanish National Cancer Research Centre (CNIO), Melchor Fernández Almagro 3, Madrid, 28029, Spain; 2 Departments of Epidemiology and Population Health & of Obstetrics & Gynecology and Women's Health, Albert Einstein College of Medicine, 1300 Morris Park Ave., New York, NY 10461, USA; 3 Queensland Institute of Medical Research, 300 Herston Road, Brisbane, 4006, Australia; 4 Division of Hematology and Oncology, David Geffen School of Medicine, University of California at Los Angeles, 10833 Le Conte Avenue, Los Angeles, CA 90024, USA; 5 Department of Laboratory Medicine and Pathology, Mayo Clinic, 200 First Street S.W., Rochester, MN 55905, USA; 6 Human Genetics Group, Human Cancer Genetics Programme, CNIO, Melchor Fernández Almagro 3, Madrid, 28029, Spain; 7 Servicio de Cirugía General y Especialidades, Hospital Monte Naranco, Avda. Dres. Fernández-Vega 9, Oviedo, 33012, Spain; 8 Servicio de Oncología Médica, Hospital Universitario La Paz, Paseo de la Castellana 261, Madrid, 28046, Spain; 9 Department of Epidemiology and Population Health, London School of Hygiene and Tropical Medicine, Keppel St., London, WC1E 7HT, UK; 10 Breakthrough Breast Cancer Research Centre, The Institute of Cancer Research, 237 Fulman Road, London, SW36JB, UK; 11 Department of Epidemiology, School of Medicine, UC Irvine, 224 Irvine Hall, Irvine, CA 92697, USA; 12 Division of Cancer Epidemiology and Genetics, Hormonal and Reproductive Epidemiology Branch, National Cancer Institute, 6120 Executive Blvd, Rockville, MD 20852, USA; 13 Department of Cancer Epidemiology and Prevention, The M. Sklodowska-Curie Cancer Center and Institute of Oncology, ul Roentgena 5, Warsaw, 02 781, Poland; 14 Centre for Molecular, Environmental, Genetic and Analytic Epidemiology, The University of Melbourne, Level 1, 723 Swanston Street, Melbourne, 3010, Australia; 15 Department of Pathology, The University of Melbourne, 5th floor, West Wing, Medical Building 181, Melbourne, 3010, Australia; 16 Radiation Epidemiology Branch, Division of Cancer Epidemiology and Genetics, National Cancer Institute, Executive Plaza South, Room 7094, Bethesda, MD 20892, USA; 17 Institute of Clinical Medicine, Department of Pathology, University of Eastern Finland, Yliopistonranta 1, Kuopio, 70210, Finland; 18 Department of Pathology, Kuopio University Hospital, Harjulantie 1, Kuopio, 70210, Finland; 19 Biocenter Kuopio, Yliopistonranta 1, Kuopio, 70211, Finland; 20 Department of Oncology, Kuopio University Hospital, Harjulantie 1, Kuopio, 70210, Finland; 21 Department of Oncology, Vaasa Central Hospital, Hietalahdenkatu 2-4, Vaasa, 65130, Finland; 22 Samuel Lunenfeld Research Institute, Mount Sinai Hospital, 600 University Ave., Toronto, ON, M5G 1X5, Canada; 23 Ontario Cancer Genetics Network, Cancer Care Ontario, 620 University Avenue, Toronto, ON M5G 2C1, Canada; 24 Departments of Molecular Genetics and Laboratory Medicine and Pathobiology, University of Toronto, 1 King's College Circle, Toronto, ON M5S 1A8, Canada; 25 Dalla Lana School of Public Health, University of Toronto, 155 College Street, Toronto, ON M5T 3M7, Canada; 26 Institute for Cancer Studies, Department of Oncology, University of Sheffield Medical School, Western Bank, Sheffield, S10 2TN, UK; 27 Academic Unit of Surgical Oncology, Department of Oncology, University of Sheffield Medical School, Western Bank, Sheffield, S10 2TN, UK; 28 Academic Unit of Pathology, Department of Neuroscience, University of Sheffield Medical School, Western Bank, Sheffield, S10 2TN, UK; 29 Department of Oncology, University of Cambridge, Strangeways Research Laboratory, Wort's Causeway, Cambridge, CB1 8RN, UK; 30 Dr. Margarete Fischer-Bosch-Institute of Clinical Pharmacology, Auerbach Str. 112, Stuttgart, 70367, Germany and University Tübingen, Wachterstrasse, Tübingen, 72074, Germany; 31 Department of Internal Medicine, Evangelische Kliniken Bonn gGmbH, Johanniter Krankenhaus, Johanniterstraße 3, Bonn, 53113, Germany; 32 Institute for Prevention and Occupational Medicine of the German Social Accident Insurance (IPA), Bürkle-de-la-Camp Platz 1, Bochum, 44789, Germany; 33 Molecular Genetics of Breast Cancer, Deutsches Krebsforschungszentrum (DKFZ), Im Neuenheimer Feld 280, Heidelberg, 69120, Germany; 34 Institute of Pathology, Medical Faculty University of Bonn, Reuterstr. 2b, Bonn, 53113, Germany; 35 Vesalius Research Center (VRC), VIB and KULeuven, Herestraat 49 B, Leuven, 3000 Belgium; 36 Multidisciplinary Breast Center, University Hospital Gasthuisberg, Herestraat 49 B, Leuven, 3000, Belgium; 37 Department of Obstetrics and Gynecology, University of Ulm, Prittwitzstrasse 43. Ulm, 89075, Germany; 38 Department of Medical Epidemiology and Biostatistics, Karolinska Institutet, Nobels väg 12A, Stockholm, SE-171 77, Sweden; 39 Human Genetics, Genome Institute of Singapore, 60 Biopolis Street, Genome, #02-01, Singapore, 138672, Singapore; 40 Kathleen Cuningham Foundation Consortium for Research into Familial Breast Cancer, Peter MacCallum Cancer Center, St Andrews Pl, Melbourne, 3002, Australia; 41 Australian Ovarian Cancer Study Group, Peter MacCallum Cancer Center, St Andrews Pl, Melbourne, 3002, Australia; 42 Cancer Epidemiology Centre, Cancer Council Victoria, 1 Rathdowne St, Melbourne, 3053, Australia; 43 Department of Epidemiology and Preventive Medicine, Monash University, Level 6 The Alfred Centre, 99 Commercial Road, Melbourne, 3004, Australia; 44 Department of Clinical Biochemistry and Department of Breast Surgery, Herlev Hospital, Copenhagen University Hospital, Herlev Ringvej 75, Herlev, 2730, Denmark; 45 Cancer Prevention Institute of California, 2201 Walnut Avenue, Suite 300, Fremont, CA 94538, USA; 46 Stanford University School of Medicine, 300 Pasteur Drive, Stanford, CA 94305, USA; 47 Department of Health Sciences Research, Mayo Clinic, 200 First Street S.W. Rochester, MN 55905, USA; 48 Division of Cancer Epidemiology, German Cancer Research Center (DKFZ), Im Neuenheimer Feld 280, Heidelberg, 69120, Germany; 49 Institute for Medical Biometrics and Epidemiology, University Clinic Hamburg-Eppendorf, Martinistrasse 52, Hamburg, 20246, Germany; 50 Department of Gynecology and Obstetrics, University Hospital Erlangen, Universitätsstrasse 21 23, Erlangen, 91054 Germany; 51 Institute of Human Genetics, Friedrich Alexander University Erlangen-Nuremberg, Schlossplatz 4, Erlangen, 91054, Germany; 52 Department of Public Health and Primary Care, University of Cambridge, Strangeways Research Laboratory, Wort's Causeway, Cambridge, CB1 8RN, UK

## Abstract

**Introduction:**

Several common breast cancer genetic susceptibility variants have recently been identified. We aimed to determine how these variants combine with a subset of other known risk factors to influence breast cancer risk in white women of European ancestry using case-control studies participating in the Breast Cancer Association Consortium.

**Methods:**

We evaluated two-way interactions between each of age at menarche, ever having had a live birth, number of live births, age at first birth and body mass index (BMI) and each of 12 single nucleotide polymorphisms (SNPs) (10q26-rs2981582 (*FGFR2*), 8q24-rs13281615, 11p15-rs3817198 (*LSP1*), 5q11-rs889312 (*MAP3K1*), 16q12-rs3803662 (*TOX3*), 2q35-rs13387042, 5p12-rs10941679 (*MRPS30*), 17q23-rs6504950 (*COX11*), 3p24-rs4973768 (*SLC4A7*), *CASP8*-rs17468277, *TGFB1*-rs1982073 and *ESR1*-rs3020314). Interactions were tested for by fitting logistic regression models including per-allele and linear trend main effects for SNPs and risk factors, respectively, and single-parameter interaction terms for linear departure from independent multiplicative effects.

**Results:**

These analyses were applied to data for up to 26,349 invasive breast cancer cases and up to 32,208 controls from 21 case-control studies. No statistical evidence of interaction was observed beyond that expected by chance. Analyses were repeated using data from 11 population-based studies, and results were very similar.

**Conclusions:**

The relative risks for breast cancer associated with the common susceptibility variants identified to date do not appear to vary across women with different reproductive histories or body mass index (BMI). The assumption of multiplicative combined effects for these established genetic and other risk factors in risk prediction models appears justified.

## Introduction

Breast cancer is known to have both a genetic and non-genetic etiology. Several common genetic susceptibility variants have recently been identified, predominantly by genome-wide association studies (GWAS). These include single nucleotide polymorphisms (SNPs) at loci containing the genes *FGFR2*, *LSP1*, *MAP3K1*, *TOX3*, *MRPS30*, *COX 11*, *SLC4A7*, and at chromosomes 8p24 and 2q35 [1-5]. To date, the only SNP associated with breast cancer risk with genome-wide statistical significance (*P *< 10^-7^) coming from candidate gene approaches is *CASP8 *[6]; more equivocal evidence has been reported for SNPs in *TGFB1 *[6] and *ESR1 *[7], among others.

It is important to determine how these common SNPs combine with other known risk factors such as age at menarche, parity, age at first birth and body mass index (BMI) [8,9] to influence breast cancer risk because this knowledge could be used to improve risk prediction models [10,11]. The identification of modification of SNP associations by other risk factors could also provide insight into the biological mechanisms by which genetic variants are implicated in breast cancer etiology. Many of these SNPs and other risk factors have been observed to be differentially associated with estrogen receptor (ER)-positive and ER-negative disease [1,4,5,7,12,13] and so interactions between them may also differ by disease subtype.

We, therefore, aimed to assess effect modification for 12 SNPs, 10 of which have been clearly associated with breast cancer risk (10q26-rs298158 (*FGFR2*), 8q24-rs13281615, 11p15-rs3817198 (*LSP1*), 5q11-rs889312 (*MAP3K1*), 16q12-rs2803662 (*TOX3*), 2q35-rs13387042, 5p12-rs10941679, 17q23-rs6504950, 3p24-rs4973768 and *CASP8*-rs17468277) and two for which there is less clear evidence of a main effect (*TGFB1*-rs1982073 and *ESR1*-rs3020314). The potential effect modifiers considered were age at menarche, ever having had a live birth, number of live births, age at first birth and BMI. A secondary aim was to evaluate these interactions in susceptibility to breast cancer subtypes defined by ER and progesterone receptor (PR) status. Data for white women of European ancestry were combined from 21 case-control studies participating in the Breast Cancer Association Consortium (BCAC).

## Materials and methods

A description of the 21 case-control studies participating in this pooled BCAC analysis is provided in Table 1, with more detailed information given in Additional Data Table S1 in Additional file 1. These included 11 population-based studies and seven studies with at least 1,000 cases and 1,000 controls. All studies collected self-reported information for cases and controls on age at diagnosis (cases) or interview (controls), racial/ethnic group (white European, Asian or other) and at least one of the following: age at menarche, ever having had a live birth, number of live births, age at first live birth (if parous), BMI (or height and weight). The time-point at which these variables were assessed for each study is detailed in Additional Data Table S1 in Additional file 1. Additional risk and other lifestyle factor information were not available at the time of the present analysis. All studies used structured questionnaires to collect these data, with the exception of the CNIO-BCS and the LMBC study, for which the information was abstracted from medical records. Nineteen studies also provided information on the ER and PR status of the tumors for a subset of cases. This information was mostly abstracted from medical records. Subjects who reported being of ethnicities other than white European were excluded, as were cases with non-invasive disease. All study participants gave written informed consent and each study was approved by the relevant local institutional review board(s).

**Table 1:** List of participating studies and number of subjects included in at least one analysis

Study Acronym	Study Name (Reference)	N(Controls)	N(Cases)	N(ER+/-)†	N(PR+/-)††
ABCFS*	Australian Breast Cancer Family Study [24]	610	1,239	701/358	731/325
BBCC	Bavarian Breast Cancer Cases and Controls [25,26]	806	1,200	719/264	640/341
BBCS	British Breast Cancer Study [27]	1,242	1,338	0/0	0/0
CGPS*	Copenhagen General Population Study [28,29]	6,555	1,450	1,088/213	505/358
CNIO-BCS	Spanish National Cancer Centre Breast Cancer Study [30]	649	351	135/49	113/87
GENICA*	Gene Environment Interaction & Breast Cancer in Germany [31]	967	917	675/194	607/260
GESBC*	Genetic Epidemiology Study of Breast Cancer by Age 50 [32]	859	573	281/182	266/188
KBCP	Kuopio Breast Cancer Project [33]	388	430	310/96	253/151
kConFab/AOCS	Kathleen Cuningham Foundation Consortium for Research into Familial Breast Cancer [34]/Australian Ovarian Cancer Study (controls only) [35]	171	323	128/63	122/48
LMBC	Leuven Multidisciplinary Breast Centre [36,37]	804	818	624/137	554/205
MARIE*	Mammary Carcinoma Risk Factor Investigation [38]	5,294	2,573	1,998/532	1,681/847
MCBCS	Mayo Clinic Breast Cancer Study [39]	1,045	1,049	764/158	673/244
MCCS*	Melbourne Collaborative Cohort Study [40]	749	682	453/170	351/272
NC-BCFR*	Northern California Breast Cancer Family Registry [41]	154	266	201/35	172/63
OFBCR*	Ontario Familial Breast Cancer Registry [41]	328	982	578/228	488/304
PBCS*	NCI Polish Breast Cancer Study [42]	2,322	1,937	1,150/597	916/827
SASBAC*	Singapore and Sweden Breast Cancer Study [43]	1,400	1,408	766/209	679/276
SBCS	Sheffield Breast Cancer Study [44]	1,088	970	458/150	170/107
SEARCH	Study of Epidemiology & Risk factors in Cancer Heredity [45]	5,282	6,352	3,438/816	1,655/848
UCIBCS*	UCI Breast Cancer Study [46,47]	465	795	512/131	436/199
USRT	US Radiologic Technologists Study [48]	1,030	696	0/0	0/0

† Number of cases with estrogen receptor (ER) positive/negative disease, respectively, if known.

†† Number of cases with estrogen receptor (PR) positive/negative disease, respectively, if known.

* Population-based case-control study.

Genotyping methods have been previously described [1,6,7,12,14]. Briefly, five studies (ABCFS, GENICA, kConFab/AOCS, MARIE and SASBAC) used Sequenom's MassARRAY system and iPLEX technology (Sequenom, San Diego, CA, USA) for most SNPs. All other genotyping was done using Taqman^® ^Assays-by-Design^SM ^(Applied Biosystems, Foster City, CA, USA). SNP *CASP8- *rs17468277 is in complete linkage disequilibrium with *CASP8-*rs1045485, which has previously been reported to be associated with breast cancer [6]. All studies included at least one blank well (containing no DNA) per 384-well assay plate, at least 2% of samples in duplicate, and a common set of 93 samples from the Centre d'Etude Polymorphisme Humain (CEPH) used by the HapMap Consortium (HAPMAPPT01, Coriell Institute for Medical Research, Camden, NJ, USA). Genotyping call rates and duplicate concordance rates were calculated after excluding samples that had previously repeatedly failed; all were greater than 95%. Concordance with CEPH genotypes was greater than 98%.

### Statistical methods

Overall genetic associations were evaluated for each of the 12 SNPs by estimating odds ratios (ORs) and their 95% confidence intervals (CI) via logistic regression, assuming multiplicative per-allele effects for the risk allele, as first reported in the literature (see Table 2). Main effects of risk factors were assessed only in the 11 population-based studies using logistic regression, adjusted for age (categorical: ≤34, 35 to 39, 40 to 44, 45 to 49, 50 to 54, 55 to 59, 60 to 64, 65 to 69, 70 to 74, ≥75 years; and continuous, the latter to account for differences between cases and controls in the extreme age-groups) and study (categorical). Risk factors considered were age at menarche (categorical: ≤11, 12, 13, 14, ≥15 years; and continuous), ever having had a live birth (no, yes), number of live births (parous women only, categorical: 1, 2, 3, ≥4; and continuous), age at first birth (parous women only, categorical: ≤19, 20 to 24, 25 to 29, ≥30 years; and continuous) and BMI, defined as weight in kilograms divided by the square of height in meters (categorical: ≤24.99, 25.00 to 29.99, ≥30.00; and continuous). Since BMI is known to be positively associated with breast cancer risk in postmenopausal women, but inversely associated with risk in premenopausal women [9], we analyzed the interactions with BMI separately for women aged <55 years and ≥55 years, considering these as a surrogates for pre- and post-menopausal status, respectively. Results from analyses using a younger age limit (50 years) to determine surrogate categories for premenopausal status were similar and are therefore not presented. Estimates of per-allele ORs for SNPs stratified by risk factors (for the categories defined above) were obtained using a single logistic regression model including appropriate dummy variables, in addition to those for the main effects of the risk factor categories.

**Table 2:** Estimated per-allele odds ratios and 95% confidence intervals for 12 SNPs, by availability of non-genetic risk factor information*

SNP	Genes at locus	**Alleles**^**†**^	**MAF**^**††**^	**OR (95%CI)**^**‡**^	**OR (95%CI)**^**‡‡ **^**based on subjects with data available on**:			
					
				All subjects	Age menarche	Parity	Age at first birth	**Body mass index**^**#**^
10q26-rs2981582	*FGFR2*	C**T**	0.38	1.22 (1.19 to 1.26)	1.22 (1.19 to 1.26)	1.22 (1.19 to 1.26)	1.22 (1.18 to 1.26)	1.21 (1.17 to 1.25)
				**(25,821/22,551)**	**(20,134/18,697)**	**(21,985/20,111)**	**(15,204/15,359)**	**(16,883/18,660)**
8q24-rs13281615	intergenic	A**G**	0.41	1.12 (1.09 to 1.15)	1.12 (1.09 to 1.16)	1.12 (1.09 to 1.16)	1.13 (1.09 to 1.17)	1.13 (1.10 to 1.17)
				**(21,823/20,609)**	**(16,779/16,698)**	**(18,060/18,081)**	**(11,808/13,610)**	**(12,969/16,531)**
11p15-rs3817198	*LSP1*	T**C**	0.31	1.08 (1.05 to 1.11)	1.08 (1.05 to 1.12)	1.08 (1.05 to 1.11)	1.09 (1.05 to 1.13)	1.08 (1.05 to 1.12)
				**(25,004/21,596)**	**(19,439/17,896)**	**(21,268/19,249)**	**(14,655/14,661)**	**(16,191/17,810)**
5q11-rs889312	*MAP3K1*	A**C**	0.28	1.11 (1.08 to 1.15)	1.10 (1.06 to 1.13)	1.11 (1.07 to 1.14)	1.09 (1.05 to 1.13)	1.09 (1.06 to 1.13)
				**(26,227/23,307)**	**(20,557/19,306)**	**(22,407/20,855)**	**(15,573/15,943)**	**(17,304/19,335)**
16q12-rs3803662	*TOX3/TNRC9*	C**T**	0.26	1.23 (1.19 to 1.26)	1.22 (1.18 to 1.26)	1.24 (1.20 to 1.27)	1.21 (1.17 to 1.26)	1.23 (1.19 to 1.27)
				**(26,132/23,459)**	**(20,628/19,334)**	**(22,478/20,874)**	**(15,613/15,966)**	**(17,356/19,351)**
2q35-rs13387042	intergenic	G**A**	0.52	1.14 (1.11 to 1.17)	1.13 (1.10 to 1.16)	1.13 (1.11 to 1.16)	1.13 (1.10 to 1.17)	1.14 (1.11 to 1.18)
				**(32,917/25,996)**	**(26,581/21,507)**	**(29,037/23,157)**	**(23,757/17,897)**	**(20,286/21,609)**
5p12-rs10941679	*MRPS30*	A**G**	0.26	1.12 (1.09 to 1.15)	1.13 (1.09 to 1.16)	1.12 (1.08 to 1.15)	1.13 (1.09 to 1.17)	1.12 (1.08 to 1.16)
				**(31,513/25,008)**	**(25,399/20,992)**	**(27,436/22,456)**	**(22,334/17,356)**	**(18,568/20,370)**
17q23-rs6504950	*COX11, STXBP4*	G**A**	0.28	0.95 (0.92 to 0.97)	0.95 (0.92 to 0.98)	0.95 (0.92 to 0.97)	0.94 (0.91 to 0.98)	0.94 (0.91 to 0.97)
				**(30,045/23,943)**	**(24,114/19,454)**	**(26,246/21,094)**	**(21,294/16,138)**	**(17,497/19,500)**
3p24-rs4973768	*SLC4A7, NEK10*	C**T**	0.46	1.11 (1.09 to 1.14)	1.10 (1.07 to 1.13)	1.11 (1.08 to 1.14)	1.10 (1.07 to 1.14)	1.11 (1.08 to 1.14)
				**(30,366/22,929)**	**(24,483/18,624)**	**(26,707/20,292)**	**(21,733/15,454)**	**(17,892/18,618)**
2q33-rs17468277	*CASP8*	C**T**	0.13	0.94 (0.91 to 0.98)	0.95 (0.91 to 0.99)	0.96 (0.92 to 0.99)	0.94 (0.90 to 0.99)	0.96 (0.92 to 0.99)
				**(32,784/25,700)**	**(26,702/21,218)**	**(29,020/22,864)**	**(23,718/17,637)**	**(20,228/21,267)**
19q13-rs1982073	*TGFB1*	T**C**	0.38	1.04 (1.01 to 1.07)	1.04 (1.01 to 1.08)	1.04 (1.01 to 1.08)	1.05 (1.01 to 1.09)	1.04 (1.00 to 1.08)
				**(24,498/17,003)**	**(19,838/14,279)**	**(20,424/14,950)**	**(17,050/11,460)**	**(11,849/13,586)**
6q25-rs3020314	*ESR1*	T**C**	0.32	1.03 (1.00 to 1.06)	1.02 (0.99 to 1.06)	1.02 (0.99 to 1.05)	1.02 (0.99 to 1.06)	1.01 (0.98 to 1.05)
				**(24,009/20,496)**	**(19,378/17,622)**	**(20,623/18,515)**	**(17,370/14,538)**	**(15,505/17,167)**

*The number of controls/cases (respectively) included in each analysis is given in square parenthesis.

^**† **^Minor allele according to first publication in bold type.

^**††**^Minor allele frequency, assessed in controls with available genotype data.

^**‡**^Odds ratio per copy of the minor allele, adjusted for study (categorical), based on all genotype data, regardless of availability of non-genetic risk factor data.

^**‡‡**^Odds ratio per copy of the minor allele, adjusted for age (categorical: ≤34, 35 to 39, 40 to 44, 45 to 49, 50 to 54, 55 to 59, 60 to 64, 65 to 69, 70 to 74, ≥75; and continuous), study and the corresponding non-genetic risk factor (continuous), based on data for subjects with genotype data and information on the non-genetic risk factor.

^**#**^Model also included an interaction term for body mass index and age (<55, ≥55).

Interaction, or modification of genetic associations by other risk factors, was assessed for each SNP/risk factor combination by fitting logistic regression models. Each model included dummy variables for study plus three parameters, one for the main per-risk-allele effect, one for the main risk factor effect (all modeled as continuous variables, except ever having had a live birth) and a single interaction term for the product of the number of risk alleles and the value of the risk factor. This was tested statistically by a likelihood ratio test comparing this model to that without the interaction term. Effect modification by BMI was assessed separately for women <55 and ≥55 years of age.

In addition, a parametric bootstrap test was used to estimate interaction *P*-values adjusted for multiple testing [15]. For each of the 72 interactions tested, we estimated the probability of being a case for each subject under the null hypothesis of no interaction, by applying the logistic regression model including only main effects for study (categorical), SNP (per-allele) and risk factor (continuous, except ever having had a live birth). Each replicate of the parametric bootstrap consisted of, for each interaction tested: (i) generating a dummy case-control status for each subject by sampling from a binomial distribution based on the estimated probability of being a case (by generating a single random number from the uniform distribution and assigning "case" to subjects for which this was less than the probability of being a case and "control" otherwise); and (ii) based on this dummy case-control status and the actual data for all other variables, fitting the interaction model described above and noting the likelihood ratio test *P*-value for the comparison of this model to the main effects only model applied to the same data. The minimum *P*-value was recorded for each of 10,000 replicates and the adjusted *P*-values were estimated as the proportion of replication *P*-values less than the corresponding unadjusted *P*-value. Results rounded to two decimal places were identical to those obtained using a standard non-parametric permutation test [15].

All statistical analyses were carried out using Stata: Release 10 (Statacorp, College Station, TX, USA) [16] with the exception of power calculations which were done using Quanto (University of Southern California, Los Angeles, CA, USA) [17,18].

## Results

The 21 participating studies contributed 26,349 cases and 32,208 controls of self-reported white European race/ethnicity, all with available data for at least one of the 12 SNPs considered and at least one of the other risk factors considered (minimal data). Of these, 17,603 cases from 18 studies (all except BBCS, MCCS and USRT) were interviewed within two years after their breast cancer diagnosis and 29,187 controls came from the same 18 studies. Forty-six percent of cases and 38% of controls were under age 55 years at diagnosis and interview, respectively. ER and PR status was known for 19,561 and 16,962 cases, respectively. Details by study are provided in Table 1. In total, 12,822 cases and 19,703 controls with minimal data were included from 11 population-based studies and 16,107 cases and 23,140 controls with minimal data were included from seven studies with at least 1,000 cases and 1,000 controls.

When analyses were restricted to population-based studies, the expected associations with breast cancer were observed for the risk factors, with one exception. After adjustment for age and study, each one-year increase in age at menarche was associated with a 4% (95% CI = 2 to 5%) decrease in breast cancer risk, and being parous was associated with a 16% (95% CI = 10 to 22%) decreased risk. For parous women, each additional live birth was associated with an 11% (95% CI = 8 to 13%) decrease in risk, while each five-year increment in age at first birth was associated with a 7% (95% CI = 4 to 10%) increase in risk. Obesity (BMI ≥ 30.0 kg/m^2^) was associated with a 20% (95% CI = 10 to 29%) lower risk of breast cancer for women under age 55 years. The one unexpected observation was that obesity was not associated with breast cancer risk in women aged 55 years and older (OR = 0.96, 95% CI 0.88 to 1.04).

Table 2 provides estimated per-allele ORs and their 95% CIs for the 12 SNPs considered, for all included subjects with genotype data, and for the subsets of women with information available for each of the four risk factors considered. All ORs were adjusted for study, and each subset was adjusted for study, age and the relevant risk factor. The OR estimates in the overall and subset analyses were very similar, and provide no evidence of confounding by the risk factors, nor of bias in OR estimates related to data availability.

For the vast majority of SNP/risk factor combinations, there was no evidence that the per-allele OR for the SNP varied by category of the risk factor. This was true for analyses based on data from all studies (Additional Data Table S2 in Additional file 1), for analyses based on population-based studies only (Additional Data Table S3 in Additional file 1) and for analyses based on the seven studies with at least 1,000 cases and 1,000 controls (Additional Data Table S4 in Additional file 1). Restricting analyses to the 18 studies with cases interviewed within two years after their breast cancer diagnosis made no substantial difference to the results obtained (data not shown). Similarly null results were observed for analyses restricted to ER-positive and ER-negative breast cancer (Additional Data tables S5 and S6 in Additional file 1) and for analyses restricted to PR-positive and PR-negative breast cancer (Additional Data Table S7 and S8 in Additional file 1).

**Figure 1:**
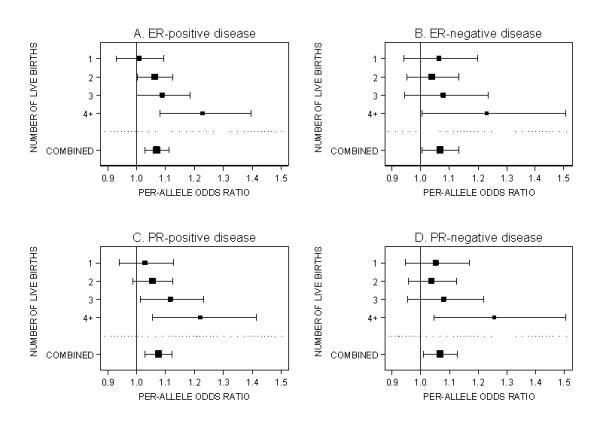
**Per-allele OR estimates for 11p15-rs3817198 (*LSP1*) stratified by number of live births *(*parous women only)**. For breast cancer disease subtypes defined by estrogen receptor (ER) and progesterone receptor (PR) status. The size of the box is inversely proportional to the standard error of the log OR estimate.

The strongest evidence of interaction (unadjusted *P *= 0.002) was for the modification of the association with 11p15-rs3817198 (*LSP1*) by number of live births. Per-allele OR estimates increased from 1.04 (95% CI = 0.97 to 1.11) for women who had had just one live birth to 1.24 (95% CI = 1.11 to 1.38) for women with at least four live births, and an interaction OR of 1.05 per live birth and per allele was estimated. This trend was also observed when data from only population-based studies and from only studies with at least 1,000 cases and 1,000 controls were considered (*P *= 0.01 in both sub-analyses). Evidence for this interaction was observed when the analysis was restricted to ER-positive and PR-positive disease (*P *= 0.004 and *P *= 0.01, respectively; Figure 1), but not for analyses based on ER-negative and PR-negative cases (*P *= 0.3 and 0.06, respectively). However, considering that 72 tests for interaction were carried out, chance cannot be excluded as an explanation for these results. The multiple-test-adjusted *P*-value for the modification of the 11p15-rs3817198 association by number of live births was 0.12. The adjusted p-values for all other interactions tested were all ≥0.61.

*Post-hoc *power calculations estimated that for age at menarche (per year), parity (per live birth) and age at first birth (per five-year age increase), our study had 90% power at a significance level of 0.0007 (corresponding to a multiple-testing-adjusted *P*-value of 0.05) to detect interaction ORs of at least 1.06 for all loci tested except *CASP8*-rs17468277, for which the minimum was 1.08. For BMI (per five-unit increase) the minimum interaction OR detectable with 90% power in both age strata (<55 and ≥55) was 1.08 for the more common variants and 1.10 for *CASP8*-rs17468277. For parity, considered as never or ever having had a live birth, the study had similar power to detect interaction ORs of at least 1.20 for *CASP8*-rs17468277 and 1.16 for the remaining loci.

## Discussion

This combined analysis of more than 25,000 cases and 30,000 controls found no conclusive evidence that age at menarche, parity, age at first birth or BMI modify the established associations of breast cancer risk with 10q26-rs298158 (*FGFR2*), 8q24-rs13281615, 11p15-rs3817198 (*LSP1*), 5q11-rs889312 (*MAP3K1*], 16q12-rs2803662 (*TOX3)*, 2q35-rs13387042, 5p12-rs10941679, 17q23-rs6504950, 3p24-rs4973768 and *CASP8*-rs17468277) nor the putative associations with *TGFB1*-rs1982073 or *ESR1*-rs3020314. This was also true for disease subtypes defined by ER and PR status.

The strongest evidence of effect modification was for number of live births and 11p15-rs3817198 (*LSP1*). However, the observed trend of increasing relative risk with increasing parity was not statistically significant after correction for multiple testing. It should be noted that the interaction OR was 1.05 per allele and per live birth. This corresponds to an estimated per-allele OR increasing from 1.04 for women with one child to 1.24 for women with four or more children, for a SNP with an estimated average OR of 1.08 across all levels of parity. Such weak interactions would only result in very small differences in estimates of joint effects relative to those from models assuming multiplicative effects. This finding in this very large study highlights the difficulty of identifying modifying effects of this magnitude.

A recent study by Travis *et al. *of 7,610 cases and 10,196 controls reported null results for interactions in breast cancer susceptibility between 9 of the same genetic loci and 10 risk factors, including age at menarche, parity, age at first birth and BMI [19]. Our null findings replicate the results from this prospective study of older women (over age 50 years), but in a study with more than twice the sample size in this age group, and confirm that they are also applicable to women under age 50 years. Our study also extends the genetic loci evaluated for interactions with a subset of established breast cancer risk factors to include 17q23-rs6504950 and 3p24-rs4973768 [1] and *ESR1*-rs3020314 [7], which were not considered by Travis *et al. *[19]. Furthermore, with regard to the susceptibility locus at 5p12, we considered the more strongly associated SNP rs10941679 rather than rs981782 (which is only weakly correlated with rs10941679) [5]. Of note, Travis *et al*. found no evidence of interaction between 11p15-rs3817198 (*LSP1*) and number of children (*P *= 0.9) [19].

One of the strengths of the BCAC is the large combined sample size achieved through international collaboration. This has proven to be very effective in confirming or ruling out association with breast cancer for common SNPs identified through GWAS and candidate gene studies [1,2,6,14,20,21]. The BCAC has also been able to provide highly precise estimates of the ORs associated with susceptibility alleles, with very high consistency observed between the many studies that participate in the consortium, despite the range of study designs represented. The inclusion of multiple studies that recruited selected cases and/or volunteer controls means that the main effects for some risk factors cannot be appropriately evaluated across the whole consortium. However, this potential selection bias in estimating main effects should not influence the assessment of interactions [22]. Nevertheless, we carried out sensitivity analyses considering only data from population-based studies and only data from studies with at least 1,000 cases and 1,000 controls and observed no substantial change in the results obtained regarding interactions. We also performed analyses of the full dataset, allowing for between-study heterogeneity in the main effects for the risk factors by including interaction terms for each, and similarly observed that this did not influence the results obtained (data not shown).

A potential limitation of our study derives from heterogeneity in data collection methods across studies. All studies except two (neither population-based) used structured questionnaires administered by a variety of means, including in-person interviews, phone-interviews and self-administration. Nevertheless, the measurement of age at menarche, ever having had a live birth, number of live births and age at first birth seem likely to be robust to these differences in data collection method. Our results for BMI may be more likely to be affected by heterogeneity in data collection methods, although standardized measurement within studies and adjustment for study as a covariate should limit this to a loss of power, rather than any systematic bias. We repeated our primary analyses excluding cases interviewed before, or more than two years after, their breast cancer diagnosis and results were not substantially different. This suggests that between-study differences in the reference time at which BMI was reported did not influence the inference from our study. A further limitation of our study was that we did not collate information on hormone therapy (HT) use from the majority of participating studies and so were unable evaluate interactions between SNPs and BMI by HT use in older women. This requires further investigation because HT has been observed to modify the effect of obesity on post-menopausal breast cancer risk [23]. Since menopausal status was not assessed and/or derived uniformly across all studies, we used age as a surrogate to more appropriately stratify analyses of effect modification by BMI. Finally, the present study had limited statistical power to detect interactions in susceptibility to ER-negative and PR-negative disease.

## Conclusions

In summary, in the largest collaborative analyses of gene-environment interactions carried out to date, we have observed no conclusive evidence for modification of the per-allele relative risk associated with common breast cancer susceptibility variants by age at menarche, parity, age at first birth or BMI. This finding is consistent with those from a recently published smaller prospective study. These results imply that the combined effects of these common susceptibility alleles and other established risk factors can be assumed to multiplicative in risk predicted models for breast cancer.

## Abbreviations

ABCFS: Australian Breast Cancer Family Study; AOCS: Australian Ovarian Cancer Study; BBCC: Bavarian Breast Cancer Cases and Controls; BBCS: British Breast Cancer Study; BCAC: Breast Cancer Association Consortium; BMI: body mass index; CEPH, Centre d'Etude Polymorphisme Humain; CGPS: Copenhagen General Population Study; CI: confidence interval; CNIO-BCS: Spanish National Cancer Centre Breast Cancer Study; ER: estrogen receptor; GENICA: Gene Environment Interaction & Breast Cancer in Germany; GESBC: Genetic Epidemiology Study of Breast Cancer by Age 50; GWAS: genome-wide association study; HT: hormone therapy; KBCP: Kuopio Breast Cancer Project; kConFab: Kathleen Cuningham Foundation Consortium for Research into Familial Breast Cancer; LMBC: Leuven Multidisciplinary Breast Centre; MARIE: Mammary Carcinoma Risk Factor Investigation; MCBCS: Mayo Clinic Breast Cancer Study; MCCS: Melbourne Collaborative Cohort Study; NC-BCFR: Northern California Breast Cancer Family Registry; OFBCR: Ontario Familial Breast Cancer Registry; OR: odds ratio; PBCS: NCI Polish Breast Cancer Study; PR: progesterone receptor; SASBAC: Singapore and Sweden Breast Cancer Study; SBCS: Sheffield Breast Cancer Study; SEARCH: Study of Epidemiology and Risk factors in Cancer Heredity; SNP: single nucleotide polymorphism; UCIBCS: UCI Breast Cancer Study; USA: United States of America; USRT: US Radiologic Technologists Study.

## Competing interests

The authors declare that they have no competing interests.

## Authors' contributions

RLM performed the statistical analyses and wrote the manuscript. MMG also carried out statistical analyses. RLM, MMG, ABS, PAF, FJC, PDP, MGC, ELG and JCC formed part of the writing group which was responsible for the interpretation of results and critical revision of the manuscript for important intellectual content. JB, NM, IdSS, OF, NJ, JF, JLH, MCS, AJS, MSL, SJS, DMF, VMK, PA, ILA, GG, JAK, HB, DL, PH, KC, JJL, GCT, LB, GS, SEB, EMJ, RH, MWB, LH and DFE also contributed to the interpretation of results and/or critical revision of the manuscript. RLM, PAF, FJC, JB, JIAP, MPZ, IdSS, LJG, HAC, AZ, LB, MES, JL, JLH, GSD, CA, MCS, AJS, MSL, DMF, VK, ILA, GG, JAK, NW, AC, MWRR, SSC, MS, AMD, HB, YDK, TB, GENICA, DL, AS, PH, JJL, GCT, kConFab, AOCS, GGG, LB, GS, SEB, BGN, HF, EMJ, DWW, ASW, CV, JEO, ZF, AV, DFJ, JH, KH, LH, MKH, JM, DFE, PDP, MGC and JCC participated in the original design, subject recruitment, acquisition of data and/or biospecimen collection for the studies that contributed data. RLM, MMG, OF, NJ, AZ, MCS, AJS, SJS, AM, ILA, AC, AMD, SA, HB, GENICA, DL, JR, SWG, AKI, HH, SEB, BGN, ZF, MK, ABE, MKH and JM carried out the genotyping and/or quality control of genotype and other data. All authors were involved in drafting the manuscript and read and approved the final version.

## Supplementary Material

Additional file 1 **Additional data tables S1 to S8**. Additional Data Table 1: Design details for each study. Additional Data Table 2: Per-allele breast cancer odds ratios (OR) estimates and 95% confidence intervals (ORlower, ORupper) for SNPs, stratified by age at menarche, ever having had a live birth, number of live births, age at first birth and BMI; based on data from all studies. Additional Data Table 3: Per-allele breast cancer odds ratio (OR) estimates and 95% confidence intervals (ORlower, ORupper) for SNPs, stratified by age at menarche, ever having had a live birth, number of live births, age at first birth and BMI; based on data from population-based studies only. Additional Data Table 4: Per-allele breast cancer odds ratio (OR) estimates and 95% confidence intervals (ORlower, ORupper) for SNPs, stratified by age at menarche, ever having had a live birth, number of live births, age at first birth and BMI; based on data only from studies with at least 1,000 cases and 1,000 controls. Additional Data Table 5: Per-allele ER-positive breast cancer odds ratios (OR) and 95% confidence intervals (ORlower, ORupper) for SNPs, stratified by age at menarche, ever having had a live birth, number of live births, age at first birth and BMI; based on data from all studies. Additional Data Table 6: Per-allele ER-negative breast cancer odds ratios (OR) and 95% confidence intervals (ORlower, ORupper) for SNPs, stratified by age at menarche, ever having had a live birth, number of live births, age at first birth and BMI; based on data from all studies. Additional Data Table 7: Per-allele PR-positive breast cancer odds ratios (OR) and 95% confidence intervals (ORlower, ORupper) for SNPs, stratified by age at menarche, ever having had a live birth, number of live births, age at first birth and BMI; based on data from all studies. Additional Data Table 8: Per-allele PR-negative breast cancer odds ratios (OR) and 95% confidence intervals (ORlower, ORupper) for SNPs, stratified by age at menarche, ever having had a live birth, number of live births, age at first birth and BMI; based on data from all studies. Click here for file
